# Development of a mathematical model to estimate intra-tumor oxygen concentrations through multi-parametric imaging

**DOI:** 10.1186/s12938-016-0235-5

**Published:** 2016-10-12

**Authors:** Chung-Wein Lee, Keith M. Stantz

**Affiliations:** School of Health Science, Purdue University, 550 Stadium Mall Drive, West Lafayette, IN 47907 USA

**Keywords:** Multi-variate Krogh model, In vivo functional imaging, Dynamic contrast enhanced computed tomography, Photoacoustic spectroscopy

## Abstract

**Background:**

Tumor hypoxia is involved in every stage of solid tumor development: formation, progression, metastasis, and apoptosis. Two types of hypoxia exist in tumors—chronic hypoxia and acute hypoxia. Recent studies indicate that the regional hypoxia kinetics is closely linked to metastasis and therapeutic responses, but regional hypoxia kinetics is hard to measure. We propose a novel approach to determine the local pO_2_ by *fusing* the parameters obtained from in vivo functional imaging through the use of a modified multivariate Krogh model.

**Methods:**

To test our idea and its potential to translate into an in vivo setting through the use of existing imaging techniques, simulation studies were performed comparing the local partial oxygen pressure (pO_2_) from the proposed multivariate image fusion model to the referenced pO_2_ derived by Green’s function, which considers the contribution from every vessel segment of an entire three-dimensional tumor vasculature to profile tumor oxygen with high spatial resolution.

**Results:**

pO_2_ derived from our fusion approach were close to the referenced pO_2_ with regression slope near 1.0 and an r^2^ higher than 0.8 if the voxel size (or the spatial resolution set by functional imaging modality) was less than 200 μm. The simulation also showed that the metabolic rate, blood perfusion, and hemoglobin concentration were dominant factors in tissue oxygenation. The impact of the measurement error of functional imaging to the pO_2_ precision and accuracy was simulated. A Gaussian error function with FWHM equal to 20 % of blood perfusion or fractional vascular volume measurement contributed to average 7 % statistical error in pO_2_.

**Conclusion:**

The simulation results indicate that the fusion of multiple parametric maps through the biophysically derived mathematical models can monitor the intra-tumor spatial variations of hypoxia in tumors with existing imaging methods, and the potential to further investigate different forms of hypoxia, such as chronic and acute hypoxia, in response to cancer therapies.

**Electronic supplementary material:**

The online version of this article (doi:10.1186/s12938-016-0235-5) contains supplementary material, which is available to authorized users.

## Background

The oxygen concentration in normal tissue is maintained in a steady state, where the oxygen diffused from the capillaries meets the metabolic demand of the surrounding cells. Disrupting this balance, such as in wound repair for extra oxygen demand, triggers physiological angiogenesis. The nascent vessel sprouting and pruning from the existing vessel network in physiological angiogenesis are tightly regulated. If the disruption is severe enough, necrosis or apoptosis develops, resulting in a stroke or myocardial infarction. Unlike physiological angiogenesis, tumor-induced angiogenesis is uncontrolled, resulting in a tortuous, leaky, and dilated microvasculature. This dysfunctional microvasculature cannot supply the oxygen demand from tumor cells, and leads to a spatiotemporal heterogeneous hypoxia distribution in tumor [1]. The hypoxia further fuels abnormal angiogenesis and disrupts other cellular mechanisms, such as immune response [2] and cell survival [3, 4]. Thus, tumor hypoxia imaging and quantification are important in cancer research and have been an active research to search better cancer therapeutic outcomes [5–8]. There are two types of tumor hypoxia: chronic hypoxia (also known as diffusion-limited hypoxia) and acute hypoxia (also known as perfusion-limited hypoxia). Recent studies demonstrate that local tumor hypoxia behavior has profound impact in tumor metastasis and therapeutic outcomes [9, 10], hence characterizing and profiling the hypoxia in high spatiotemporal resolution can not only explore the further understanding causality between hypoxia and tumors but also is beneficial to cancer patient care.

Current in vivo preclinical diagnostic methods to determine the partial pressure of oxygen (pO_2_) include polarographic electrode (or Eppendorf probe), photoluminescence-quenching optical probe or biomarker assays based on the 2-nitroimidazoles (pimonidazole and EF5). These techniques provide a direct and quantifiable measure of pO_2_ or the relative oxygen level (pO_2_ < 10 mmHg) or hypoxic fraction in tissue, but their disadvantages have limited their wide spread use. For example, the Eppendorf probe necessitates an invasive procedure (needle insertion or biopsy) and lacks spatial information. Oxygen level quantification using 2-mitroimidazoles result in inconsistencies with Eppendorf probe measurements [11, 12] or with locoregional tumor control, disease-free survival [13] and event-free survival time [14]. PET and SPECT radiopharmaceutical tracers engineered around these biomarkers, such as ^18^F-MISO or ^64^Cu-ATSM, provide a noninvasive means to measure hypoxia [15, 16], and have been found to correlate with therapeutic response and outcome from radiation and chemotherapy [17, 18] when comparing average tumor-to-muscle ratios. However, these methods are still in the early stages of research and lack any mechanistic information causal to hypoxia.

Other approaches first quantify the hemodynamic parameters then extrapolate the local oxygen concentration. Near-infrared spectroscopy, such as diffuse optical tomography and photoacoustics, is widely used to measure hemoglobin concentration (C_tHb_) and its oxygen saturation (SaO_2_) level by quantifying the differential absorption spectrum of oxy- and deoxy-hemoglobin molecules [19, 20]. Quantitative BOLD MRI is also capable of measuring oxygen saturation levels in vivo as demonstrated in preclinical studies [21] and have been shown to correlate to pimonidizole staining. The oxygen concentration using dynamic contrast enhanced imaging by injecting contrast agents in blood circulation to measure local vascular hemodynamics correlates to Eppendorf pO_2_ histograph and pimonidizole-based immunohistochemistry [22, 23]. Each of these techniques provides a quantifiable measure of a single factor contributing to hypoxia, such as perfusion, hemoglobin concentration, or oxygen saturation, with good spatial and temporal resolution. By combining or fusing these parameters through the implementation of oxygen transport models, the precise local pO_2_ and etiology leading to hypoxia can be investigated. Integration of mathematical modeling and imaging observations proves to be a powerful approach to understand tumor vasculature behaviors at various scales and can have application in clinical diagnosis [24–26]. The approach will become more reliable as the progression of imaging hardware provides accurate anatomical and functional measurements in high spatiotemporal resolution.

The advent of mathematical modeling in oxygen transport began as early as the twentieth century. Krogh in collaboration with Erlang, a mathematician, first approached the modeling of oxygen transport as a frame work to design experiments, interpret data, and suggest new insights on how the smallest micro vessels can supply oxygen to tissue, given the lack of technology at the time in support of his quest to understand oxygen transport in capillaries. With his model, he was able to conclude that the skeletal muscle tissue milieu was already well oxygenated and that the capillary bed only exchanged very low amount of oxygen with the muscle cells, contrary to the common belief at the time [27]. Krogh’s tissue cylindrical model set the stage for almost all subsequent models and has had tremendous and continuous impact to the field. By the 1960s, an interest in tissue oxygen transport was renewed, and steady-state and time-dependent models were developed to investigate the influence of hemoglobin and myoglobin oxygen binding within the microcirculation and whole in organs systems [28]. These models expanded upon Krogh’s model to include compartments for the RBCs, the plasma layer, the capillary wall, intestinal space and cellular volume, and used to study muscle physiology [29]. For example, Hellums used these concepts of mass transport to predict the passage of RBCs through capillaries would result in pO_2_ fluctuations [30], a phenomenon only recently observed [31, 32]. Multiple parallel vessel models better accounted for the O_2_ diffusion between capillaries, where advanced numerical techniques were beginning to applied, including finite difference and finite element methods [33]. When combined with advanced computational systems, local oxygen concentrations due to tissue heterogeneity could be further investigated [33]. By 2000, Secomb, Hsu, and Pries developed a Green’s function algorithm to calculate the steady-state local pO_2_ contributed from all vessel segments within a 3D network [34, 35], and was used to study the development of microvascular networks as well as different regulatory mechanisms on blood flow in tumors [36–38]. By implementing finite difference methods, time-dependent solutions of heterogeneous microvascular networks under various physiological conditions were now possible [39].

**Fig. 1:**
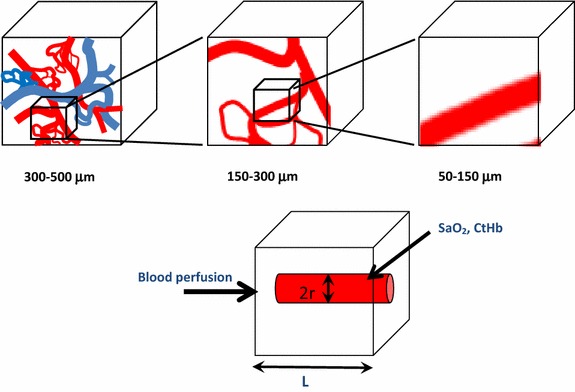
Schematic illustration of the vascular reduction as function of the voxel size. As the voxel size is reduced, the vascularity of the regional vessel network within the voxel becomes less complex; thus, using a single effective vessel with multi vascular attributes to calculate the local pO_2_ becomes possible. In the MPO2 model, a single straight cylindrical vessel is placed at the center of the voxel and the average voxel pO_2_ (MPO2) is calculated. The effective vascular structure and its functional inputs (e.g. blood flow, hemoglobin concentration, and fraction of vessel volume) are obtained from in vivo imaging measurements, thus providing a means to fuse this information based on the biophysics model approach

The objective of this study is to profile the 3-D oxygen concentration and hypoxic fraction by fusing the multiple parameters obtained from in vivo functional imaging through the use of oxygen transport model. To obtain local oxygen concentration levels based on 3-D imaging, the Krogh model has been reformulated, where the inputs are obtained from the parametric images of the vascular physiology and hemoglobin status within the tumor. Thus, a multivariate image-fusion model of pO_2_ (or MVIF model) is provided. The MVIF model depends on the spatial resolution of the imaging system, as the point spread function decreases, the voxel volume will contain a single vessel and the local pO_2_ level will be determined with higher accuracy and precision, see Fig. 1. Prior to in vivo testing, simulation studies are performed to investigate the viability of the MVIF model, where previously published biological models and microscopic data were used as a point of reference. The goals of this study are to identify an optimal voxel size and the necessary instrumental sensitivities to obtain an accurate and precise determination of pO_2_. The range of spatial resolutions and sensitivities used in these simulation studies are consistent with in vivo dynamic contrast-enhanced (DCE) imaging (clinical and preclinical MRI, CT, or photoacoustic) and optical or photoacoustic spectroscopy (PCT-S). 3D tumor vasculature and hemodynamic quantification using intravital microscopy are used to simulate the in vivo parametric measurements (such as perfusion, fractional plasma volume, and the hemoglobin status within each voxel) which are subsequently fused to our model to obtain 3D pO_2_ maps. These MPO2 maps are compared on a voxel-by-voxel basis to the referenced pO_2_ values calculated by Green’s function [34]—which shows close correlation to the local tumor oxygen level using microelectrode [40]—to investigate the necessary image resolution and to determine the sensitivity by varying the statistical and systematic uncertainty in the in vivo imaging parameters.

## Methods

### Simulation procedure overview

**Fig. 2:**
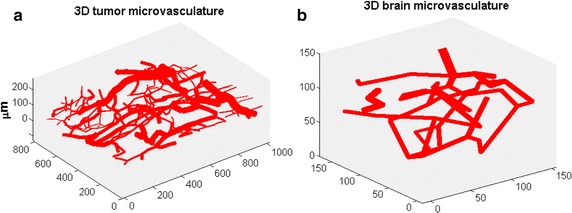
Illustration of reconstructed three-dimensional microvessel network of a **a** tumor (1000 × 1000× 150 μm^3^) and **b** brain (150 × 160 × 140 μm^3^) by confocal scanning microscope with sub-μm resolution (all axis’s are in units of micrometers)

The performance of our model was tested in two different tissue types: the brain and a tumor. The microvasculature within the brain served as an internal control or normal tissue, while the microvasculature of the tumor represented an abnormal or diseased tissue. The 3-D microvasculature of the tumor and the brain is displayed in Fig. 2a and b, the 3D dimension of tumor encompassing entire vasculature was 0.990 × 0.810 × 0.15 mm^3^, the dimension of brain was 0.150 × 0.160 × 0.14 mm^3^. Basically a window chamber was employed into the dorsal flap of the animal and tumor cells were implanted subcutaneously near a vessel in the chamber to study tumor-induced angiogenesis. Intravital microscopy was used to image 3D tumor vascular structure; photometric techniques were used to quantify hemodynamic variables (such as blood flow, hematocrit) in each vessel segment of the vasculature. The detailed experimental protocol and the procedures of post image and video processing to derive blood flow and vascular branching angle can be found in the publication of Fontanella et al. [41] and Brizel et al. [42]. The Green’s function algorithms to utilize the experimental vascular inputs from window chamber model in calculating oxygen distribution were downloaded from the website [43].

In this study, the oxygen profile calculated by Green’s function was used as a reference. The Green’s function method calculates the pO_2_ at any location within the tumor by considering the contribution from all single vessel segments in the tissue volume. Once the pO_2_ profile within the entire tissue volume (brain or tumor) was completed, a virtual 3-D voxel grid representing the reconstructed image volume by DCE-CT was superimposed onto the tissue. The average effective hemodynamic values in each finite voxel were calculated based on its known microvascular architecture. These voxel-based hemodynamic parameters are used as inputs to our single-vessel multivariate oxygen transportation model (MVIF) to calculate the pO_2_ (MPO2) in each voxel of the virtual grid. The corresponding pO_2_ (GPO2) calculated using Green’s function was also calculated and used as a reference value. A scatter plot of MPO2 versus GPO2 for each voxel was compared by performing a linear regression. Since the placement of the virtual (or imaging) voxel grid is somewhat arbitrary and depends on the voxel size, the grid was randomly translated by less than a half a voxel length for larger voxel sizes. To account for these variations and to increase the number of data points for large-voxel MPO2 to GPO2 data, the grid was translated, the new vascular architecture for each voxel extracted, and the MPO2 and GPO2 calculated and plotted.

### Implementing Green’s function algorithm to calculate referened pO_2_ (GPO2)

**Fig. 3:**
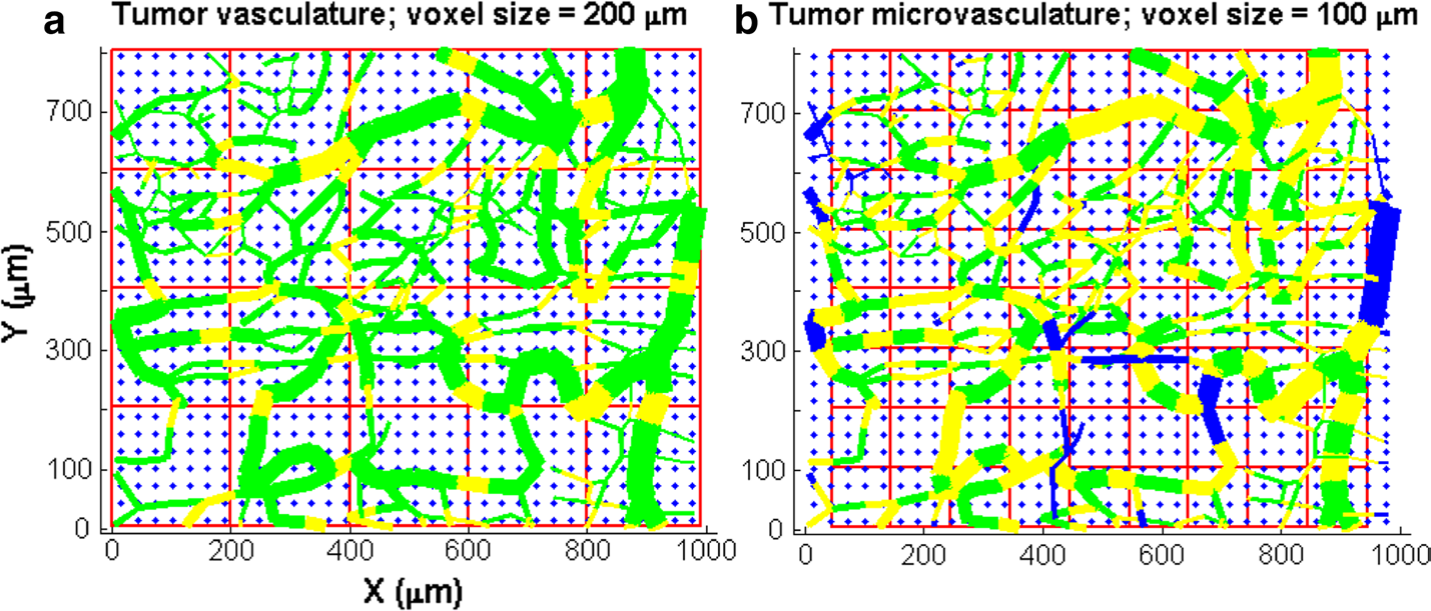
A 2-D schematic of a cubic lattice used to evaluate the detailed pO_2_ profile in a tumor and the virtual grid to simulate the voxels from in vivo functional imaging modality. **a** The cubic lattice structure (*blue diamonds*) is used to calculate the pO2 value using Green’s function algorithm, where the edge length of the lattice is 15 µm. A 200-µm virtual grid is superimposed over the tumor. The color of the vessel segment indicates the geometrical relation of the vessel segment to the voxel. *Green vessel* segments are located entirely within the voxel; *yellow segments* represent vessels that cross two voxels; and blue segments are located outside the grid. **b** An example of a 100-µm virtual grid of the same tumor is displayed for comparison

The Green’s function algorithm developed by Hsu and Secomb [34] was used to calculate the tissue oxygen concentrations resulting from the unique microvasculature geometry and hemodynamics in each tissue. The physiological constants used in these simulations were listed in Table 1. To reduce computation time, the tissue space was first discretized into an isotropic cubic lattice, the distance between two lattice points was 15 μm in our simulation (see Fig. 3). The oxygen consumption rate at each lattice point was derived from the Michaelis–Menten equation. Similarly vessels as oxygen source were discretized into sub vessel segments with 50 μm in length. The midpoint of each sub vessel segment represented the location oxygen source. In Green’s function algorithm, the major oxygen release factors (blood flow, oxygen diffusion rate in vessel and in tissue space, hemoglobin concentration, and oxygen release rate from hemoglobin into plasma simulated by Hill’s equation) and oxygen consumption rate were considered to calculate pO_2_ at the tissue lattice point (LPO2). Although Green’s function algorithm assumes the system to be under steady-state conditions and other assumptions to reduce the computational complexity, a statistically significant correlation between the tumor oxygen consumption rate derived by Green’s function and the actual microelectrode measurements in microvasculatures was reported [40], which provides a measure of confidence in pO_2_ from Green’s function as a reference.

**Table 1:** Key simulation parameters used in Green function model

		Tumor	Brain
1	Maximum oxygen consumption rate, *M*_*0*_	0.0004 cm^3^/cm^3^/sec	0.0025 cm^3^/cm^3^/sec
2	Total blood inflow	230.37 nL/min	10.8 nL/min
3	Saturated hemoglobin concentration $C'$	8800 μM	8800 μM [50]
4	*n* in Hill equation	3.0	3.0 [34]
5	$P_{50}$	26 mmHg	26 mmHg [50]
6	Oxygen diffusion constant *D*	2000 μm^2^/s	2000 μm^2^/s [63]
7	Oxygen solubility in blood	0.0385 μl/g mmHg	0.0385 μl/g mmHg [64]

Once all LPO2 was determined, a virtual grid composed of isotropic finite-sized voxels was superimposed onto the entire tissue, for example Fig. 3a and b demonstrated the virtual grid of 100- and 200-μm voxel overlaid on tumor. The voxel pO_2_, referred to as GPO2, was calculated by averaging all the LPO2 located inside the voxel, and represented the reference to evaluate the voxel pO_2_ by our multivariate single-vessel model.

### Implementing the multivariate image-fusion (MVIF) model to calculate voxel pO_2_(MPO2)

All MVIF algorithms were programmed in Matlab (Mathworks Inc.) A detailed derivation of the multivariate image fusion model of oxygen concentration (MVIF) is described in the Additional file 1. MVIF is a modified single cylindrical vessel model proposed by Krogh and Erlang [27, 44] used to calculate the pO_2_ in a voxel of tissue based as a function of several key physiological parameters related to oxygen supply and consumption. The final analytic equation of MVIF to calculate pO_2_ in cylindrical coordinates is:

$$pO_{2} (r,z) = pO_{2} (z = 0) - \frac{M \cdot H}{(1 + m) \cdot F}G_{2} (z;r_{c} ,r_{T} ) - \frac{M \cdot H}{2D}G_{1} (r;r_{c} ,r_{T} ).$$

*F* represents blood perfusion, $r_{c}$ is the single vessel radius, $r_{T}$ is the upper radial boundary in MVIF, $pO_{2}\,(z = 0)$ is the initial pO_2_ at the entrance of the vessel, $D$ is the oxygen diffusion constant, $H$ is Henry’s constant, $M$ is the oxygen consumption rate, $m$ is the slope of the oxygen-hemoglobin dissociation curve (see equation 2 in the Additional file 1), $G_{1}$ is the geometrical term or form factor depicting radial diffusion within the voxel, and $G_{2}$ is the geometrical term or form factor of advection along the z of cylindrical vessel.

In this simulation study, since the vasculature geometry and hemodynamic measurement inside the voxel of the virtual grid are known, some inputs for MVIF—such as the effective vessel radius $r_{c}$, blood perfusion $F$, and the initial pO_2_—can be calculated precisely as they were to measure by functional imaging modalities. Hill’s equation was used twice to calculate the initial pO_2_ in each voxel in this simulation. The lumen pO_2_ in each vessel segment of the vasculature was calculated using Green’s function, the corresponding SaO_2_ in each vessel segment, therefore, can be derived using Hill’s equation. In simulation the vessel segment ID and volume contribution within each voxel were recorded, the voxel SaO_2_ from all vessel segments within the voxel was calculated using the following formula:$\sum\limits_{i} {{\text{SaO}}_{2} (i)*{\text{vol}}(i)/V}$, ${\text{SaO}}_{2} (i)$ is the oxygen saturation of vessel segment i; ${\text{vol}}(i)$ is the volume of vessel segment i within the voxel; $V$ is the total vascular volume of the voxel. We used Hill’s equation again to derive the initial pO_2_ as input for MVIF from this voxel SaO_2_. The parameters or constants appearing in MVIF and Hill’s equation were listed in Table 2. The pO_2_ at any point within the voxel vessel was calculated; the average pO_2_ from the pO_2_ within the voxel represented the voxel pO_2_ calculated by MVIF, referred to as MPO2. Scattering plot of MPO2 versus GPO2 (as reference) on a voxel-by-voxel basis was plotted to evaluate the correlation and to identify the voxel outlier of which MPO2 significantly deviated from GPO2.

**Table 2:** Key simulation parameters used in MVIF model

		Tumor	Brain
1	Maximum oxygen consumption rate, *M*_*0*_	0.0004 cm^3^/cm^3^/sec	0.0025 cm^3^/cm^3^/sec
2	Saturated hemoglobin concentration $C'$	8800 μM	8800 μM [50]
3	$P_{50}$	26 mmHg	26 mmHg [50]
4	*n* in Hill equation	3.0	3.0 [34]
5	Henry’s constant, *H*	0.74 mmHg/μM	0.74 mmHg/μM [65]
6	Oxygen diffusion constant *D*	2000 μm^2^/s	2000 μm^2^/s [63]
7	Initial pO_2_ determination (see “Method” section)	Convert voxel SaO2 using Hill’s equation [64]	
8	Effective vessel diameter (see “Method” section)	By all vessel segments within the voxel	
9	Effective voxel blood flow (see “Method” section)	By all vessel segments within the voxel	
10	Effective SaO_2_ (see “Method” section)	Convert intravessel pO_2_ to SaO_2_ using Hill’s equation	

### MVIF response to voxel size (image spatial resolution)

In general, the complexity of the vessel network depends on the voxel size and the tissue type, as illustrated in Figs. 1, 2 and 3. In functional imaging image spatial resolution is determined by the voxel size. To investigate the accuracy and precision of the MVIF model as function of the voxels sizes, linear regression analysis was used to evaluate the correlation between MPO2 and GPO2 at different voxel sizes ranging from 50 to 300 µm, in 50 µm increments. For the brain tissue, only 50 and 100 µm voxel sizes were investigated because of the size of the tissue volume, 150 µm × 160 µm × 140 µm.

### Hypoxic fraction correlation

The hypoxic fraction (HF) is the fraction of the tumor volume that has a pO_2_ less than a defined threshold, which typically ranged between 2.5 and 10 mmHg. Tumor HF has been used in clinical diagnosis, and has been estimated by a variety of techniques including immunohistochemistry, computed tomography, or [^18^F] 2-fluoro-2-deoxy-glocose PET [23, 45]. Similarly, the relationship between the HF as determined from MPO2 and GPO2 models was investigated. First, the tumor HF for MPO2 and GPO2 was calculated for various thresholds, from 2.5 to 15 mmHg in 2.5 mm steps. The number of voxels exceeding the hypoxic threshold was counted and divided by the total number of voxels in tumor. Since the brain (or normal) tissue is well oxygenated, a pO_2_ threshold ranging from 16 to 22 mmHg with 2 mmHg intervals was used.

### Sensitivity and error analysis in MVIF modeling

Since nonlinear MVIF takes the hemodynamic and structural inputs from in vivo functional imaging to calculate MPO2, it is important to know (1) its response to changes in the input parameters and (2) how the hemodynamic or structural measurement error from in vivo functional imaging experiment affects the accuracy and precision of MPO2.

To answer the first objective the referenced MPO2 was calculated with the input values listed in Table 2. We then calculated MPO2 when only one input was considered as variable while other inputs remained as constant. Four new inputs which are ±10 and ±20 % from the referenced input value listed in Table 2 were used to calculate the corresponding MPO2 responses. The percent change in MPO2 compared to the referenced MPO2 value was then calculated and plotted. In this study we tested the MVIF response to following inputs: perfusion, vessel radius which is proportional to the fractional plasma volume, concentration of hemoglobin (C_tHb_), oxygen saturation (SaO_2_), and maximum oxygen consumption rate (M_0_). The reference values for blood perfusion, C_tHb_, and the SaO_2_ were selected from DCE-CT and PCT-S measurements [46–49], and the maximum oxygen metabolic rate was chosen from the literature [50].

The second objective aims to learn how much the potential measurement error of functional DCE-CT could impact on the MVIF pO_2_ prediction in accuracy and precision. For this purpose we chose the voxel size equal to 150 μm because the scattering plot of GPO2 vs. MPO2 showed good correlation (see Fig. 5). Since blood perfusion and the fraction of vessel volume are two key DCE-CT measurements to tissue oxygenation, we evaluated the measurement error influence of those two major hemodynamic parameters to MVIF model. A Gaussian error function was used with a full-width at half-maximum (FWHM) equal to 20 % of the blood perfusion to generate ten mock blood perfusions for each voxel, and then the corresponding ten MPO2 were calculated. The average of ten MPO2 and the standard error were calculated and plotted as a function of GPO2. Identical procedures were applied to the fractional vessel volume to investigate how the error in the fraction of vessel measurements from DCE-CT could impact the MVIF performance.

### Nearest-neighbor algorithm to reduce the outliers at 50-μM voxel scattering plot

The 50 μm voxel size for the tumor resulted in a significant increase in the variance and deviation from a slope of 1.0 as determined from the regression analysis (see Fig. 5a). This was not observed in the normal brain microvasculature. Unlike the brain, as the voxel size decreased for the tumor, an increasing number of voxels did not include a blood vessel, included a very small section of a blood vessel, or had a neighboring voxel with a large blood vessel. To compensate for the influence of the 6-neighboring voxels on a voxel’s average pO_2_ value, the average MPO2 from these surrounding voxels was added to the MPO2 value from the voxel itself to improve the correlation at 50 μm, or equivalently, to incorporate (or approximate) the boundary conditions. Therefore, the MPO2 calculation in each voxel was replaced by the average of surrounding six voxel pO_2_.

## Results

### Correlation between MPO2 And GPO2 as a function of voxel size

**Fig. 4:**
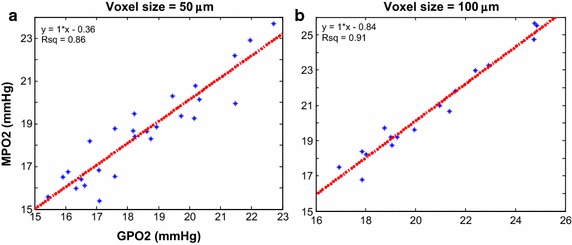
Correlation between MPO2 and GPO2 in the brain. **a** Displayed is the *scatter plot* and linear regression fit for a **a** 50 μm and **b** 100 μm voxel size. The regression results (slope, offset, and r^2^) are shown in the *top left corner*

**Fig. 5:**
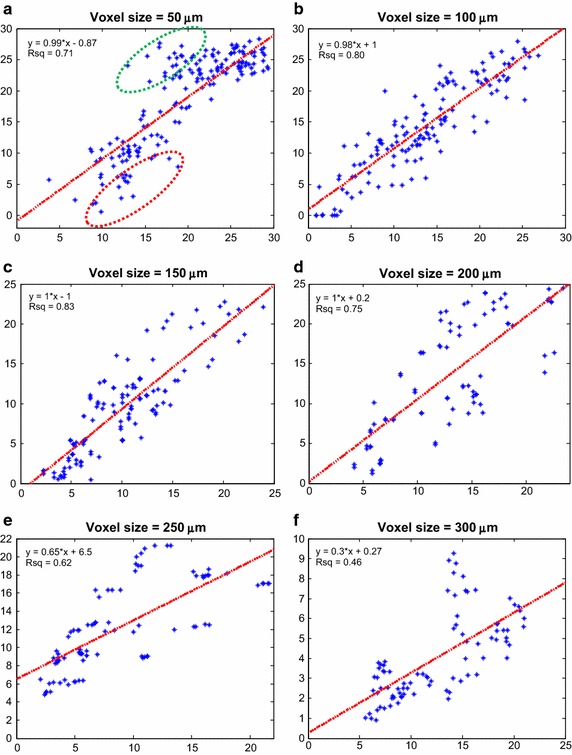
Correlation between MPO2 and GPO2 in the tumor at various voxel sizes. At 50-µm voxel size (**a**), two outlier populations (highlighted by *green* and *red dash ovals*) were observed. The *green oval* indicates the voxels that were overestimated, MPO2 significantly greater than GPO2, and the red oval the voxels that were underestimated, MPO2 significantly lower than GPO2. The correlation and linear regression analysis for voxel sizes 100, 150, and 200 μm (see **b**–**d**) are better compared to the other voxel sizes (see **a**, **e**, **f**)

For the normal brain microvasculature, the scatter plots of the average Green’s function pO_2_ (GPO2) and multivariate image fusion model pO_2_ (MPO2) for voxel dimensions of 50 and 100 µm were plotted in Fig. 4. At 50-μm voxel, the (slope, r^2^) values using linear regression analysis were (1.0, 0.86), at 100-μm voxel, the (slope, r^2^) were (1.0, 0.97). For the abnormal tumor microvasculature, the scatter plot and linear regression analysis for the six different voxel sizes (50–300 μm in 50 μm increment) were plotted in Fig. 5a–f. The (slope, r^2^) using linear regression analysis at voxel sizes from 50 to 300 μm in 50 μm increment were (0.99, 0.71), (0.98, 0.80), (1.0, 0.83), (1.0, 0.75), (0.65, 0.62), and (0.3, 0.46). The corresponding Pearson correlation coefficients (R) were 0.84, 0.89, 0.86, 0.84, 0.72 and 0.67 respectively, and all correlation coefficients reached statistical significance (p < 0.05).

**Fig. 6:**
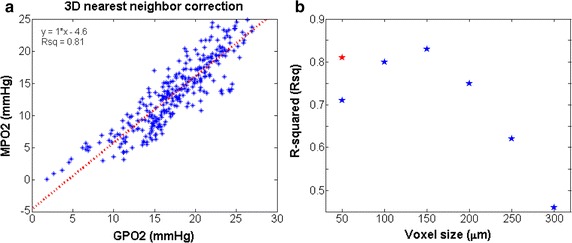
Correlation between MPO2 and GPO2 in the tumor after applying the nearest-neighbor algorithm for the 50-µm voxel data. **a** The scatter plot and linear regression fit are displayed after applying the nearest neighbor algorithm (see Fig. 5a for comparison). **b** The distribution of r^2^-values as a function of voxel size. The blue stars are from the r^2^-values in Fig. 5 and the red star is after applying the nearest neighbor algorithm to the 50-μm voxel data

In Fig. 5a–c, the r^2^ value for the 50 μm voxel size was lower than that of 100 and 150 μm, which can be explained by the influence of oxygen contribution from the vessels (arterioles or venules) in the neighboring voxels. The nearest neighbor algorithm was applied to approximate this effect, where the average pO_2_ from the surrounding voxels was added to the MPO2 value and accounts voxel lacking vasculature and outliers observed in the scatter plots. After applying this algorithm, the correlation between MPO2 and GPO2 at 50 μm significantly improved (compare of Figs. 5a, 6a). The r^2^ as function of voxel size were displayed in Fig. 6b.

### Hypoxic fraction correlation between MPO2 And GPO2 as function of voxel size

**Fig. 7:**
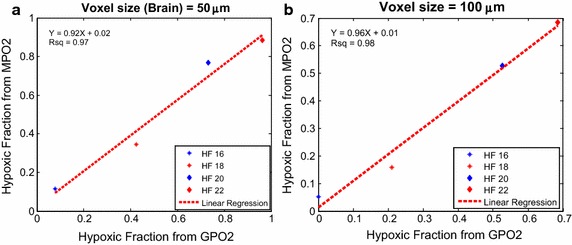
Normal brain tissue hypoxic fraction (HF) correlation of MPO2 and GPO2 in voxel sizes at 50 and 100 µm plotted in subplot **a** and **b**. In each voxel size, four HFs (16–22 mmHg in 2 mmHg increment) is used for linear regression analysis. The linear regression is plotted in *red dash line*; the result of linear regression and the Rsq values are displayed in *top left corner*

**Fig. 8:**
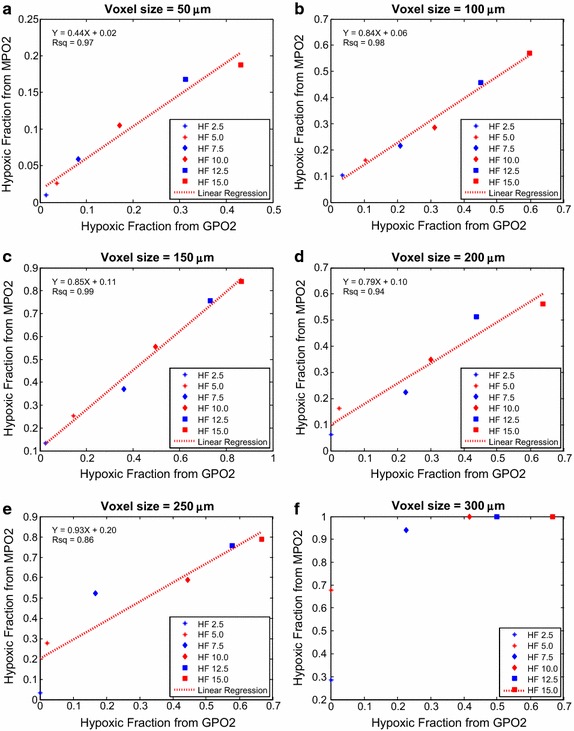
Tumor hypoxic fraction (HF) correlation of MPO2 and GPO2 in voxel sizes from 50 to 300 µm in 50 µm increment is plotted in subplot **a**–**f**. In each voxel size, six HFs (2.5–15 mmHg) is used for linear regression analysis. The linear regression is plotted in *red dash line*; the result of linear regression and the Rsq values are displayed in *top left corner*

Hypoxia fraction (HF) of tumor is another common clinical relevance parameter to evaluate tumor oxygen level. In the brain, the pO2 threshold values were set to 16, 18, 20, and 22 mmHg; and in the tumor, the thresholds were set to 2.5–15 mmHg in 2.5 mmHg increment. Figure 7 demonstrated the normal brain HF correlation and regression analysis of GPO2 and MPO2 model; the (slope, r^2^) for voxel size of 50 and 100 μm were (0.92, 0.97) and (0.96, 0.98) respectively. Figure 8 showed the tumor HF correlation and linear regression of voxel size 50–300 μm in 50 μm increment; the respective (slope, r^2^) of linear regression for voxel size from 50 to 250 μm in 50 μm increment were (0.44, 0.97), (0.84, 0.98), (0.85, 0.99), (0.79, 0.94) and (0.93, 0.86).

### Sensitivity of MVIF To microvascular hemodynamic and structural inputs

**Fig. 9:**
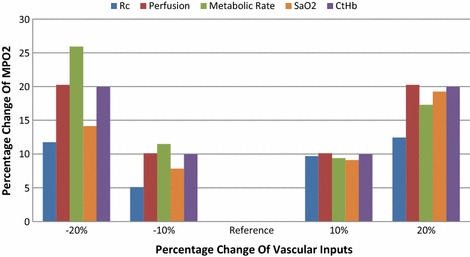
Sensitivity of MPO2 to the vascular input arguments. To investigate the systematic effects of changing the vascular arguments on pO_2_ in the tumor voxels, the vessel radius (Rc), blood perfusion, metabolic oxygen consumption rate, oxygen saturation rate (SaO_2_), and hemoglobin concentration (C_tHb_), are varied by −20 %, −10, 10, and 20 % from their respective reference values (Table 1). The percent deviation in the MPO2 relative to the original value (listed in Table 1) is plotted

The changes in the local pO_2_, either GPO2 or MPO2, due to systematic offsets in the vascular inputs are displayed in Fig. 9. Those vascular inputs were chosen based on the reported influence on tumor oxygenation [51, 52], and determined from DCE-CT or PCT-S measurements [46–49]. These values were 10 and 20 % change in the blood vessel radius (thus, fractional vascular volume), blood perfusion, oxygen metabolic rate, oxygen saturation (SaO_2_), and hemoglobin concentration (C_tHb_) relative to the reference values (Table 1). The absolute fluctuation in the average MPO2 from the four vascular inputs was 9.7, 15.2, 16.0, 12.6, and 15.0 %, respectively. The oxygen consumption rate had the largest impact on tissue oxygenation, while the vessel radius (or the fractional vascular volume) had the lowest impact on tissue oxygenation.

### MVIF response to the measurement error of microvascular inputs

**Fig. 10:**
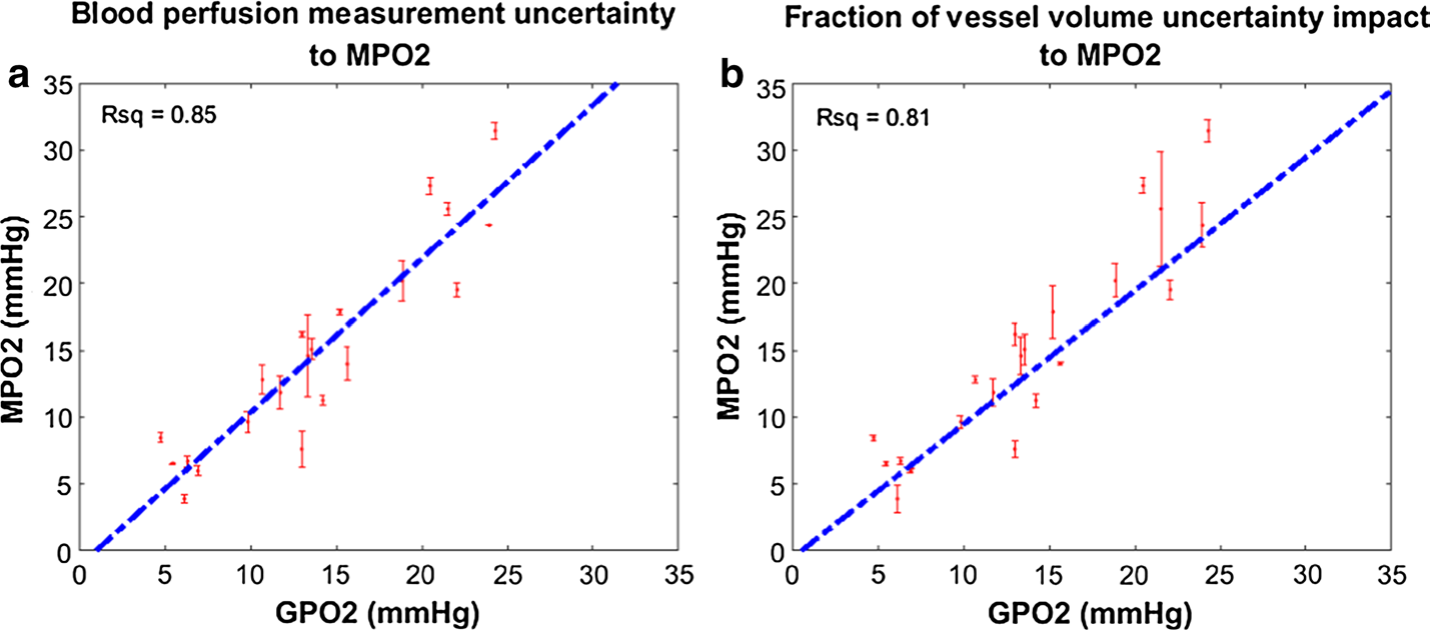
The error in MPO2 due to the measurement uncertainty in the vascular input arguments. Plotted is the pO2 using MPO2 model versus GPO2 on a voxel-by-voxel basis, using the 100 μm voxel data. **a** Displayed is the simulated fractional vessel volume including the added noise using a Gaussian function (20 % noise at FWHM), and (**b**) and for perfusion

To investigate how the precision of the measurement using in vivo functional imaging modality (e.g. DCE-CT) influence the MPO2 accuracy and precision, a Gaussian error function was used to generate ten configurations of perturbed maps of blood perfusions fractional vessel volume and the MPO2 and GPO2 maps calculated. The full width at half maximum (FWHM) of the Gaussian error function was set to 20 % of the mean vascular input value. The variation in MPO2 due to the measurement error in perfusion for the 150 μm voxel size is shown in Fig. 10a, where the error bar represents the standard deviation from these ten simulations. Similarly, the MPO2 variation due to the measurement error in the fractional vessel volume is shown in Fig. 10b. The Coefficient of variation (CV) is used to quantify the dispersion of the data relative to the mean. An average CV of the MPO2 as a result of the measurement error in blood perfusion and the fraction of vessel volume was 6.4 and 7.0 %.

## Discussion

Many preclinical and clinical cancer investigations suggest that two types of hypoxia occur heterogeneously within a solid tumor: acute hypoxia (cycling hypoxia) and chronic hypoxia (diffusion-limited hypoxia) [10, 53]. In several tumor model acute hypoxia promotes tumor angiogenesis and tumor metastasis [10, 54], hence determining the etiology of hypoxia can provide new insights into predicting therapeutic response and developing novel therapeutic protocols to enhance efficacy. Here we proposed a novel assay to delineate diffusion- and perfusion-based hypoxia based on insufficient vasculature related to (G_1_ and D, the geometric diffusion length and rate, respectively) and poorly blood perfused tissue related to (G_2_ and F, the geometry related to convective forces and perfusion) under a steady-state conditions (see equation  3 and 4 in Supplement Data). By considering the variation of these parameters as a function of time [e.g., Perfusion = Perfusion(t) and m = m(t)], acute and chronic hypoxia could be assessed, which could be tested relative to variations in angiogenic (VEGF, FGF, etc.) and inflammatory (IL6, histamine, prostaglandins, NO) activity or their corresponding therapies. As an initial step, an in vivo high-resolution oxygen profiling assay is being proposed to image the spatial variations in tumor hypoxia. This capability will further advance our understanding the role hypoxia has in solid tumors as well as in developing new cancer treatments, such as in anti-angiogenic therapy.

In this study, a method was proposed that *fuses* microvascular functional attributes from functional imaging modalities through a multivariate oxygen transport model to obtain local tumor oxygen levels was investigated. High-resolution DCE-CT (or DCE-PCT) and PCT-S can longitudinally characterize local vessel structure and hemodynamics as well as tumor metabolic status. Those measurements represent tumor oxygenation status thus in principle a biophysical oxygen transportation model, in this case a multivariate modified Krogh model for a finite volume, can take the real measurements as inputs to calculate local oxygen level, and furthermore to profile tumor oxygen status. The major challenge to verify the model prediction is to have simultaneous tumor functional measurements and corresponding oxygen level measurement in the finite volume. To the best of our knowledge, no such experiment or data has been conducted or published. To evaluate this idea and our multivariate model, we used the well characterized tumor or brain microvasculature and functions using intravital confocal microscopy, and Secomb’s 3D oxygen transportation model to calculate the pO_2_ distribution as a reference to evaluate the voxel pO_2_ from MVIF model. The tumor oxygen consumption rates derived from Secomb’s 3-D model agree closely with the oxygen electrode probe measurements [40]. A major limitation of this study is the limited photon penetration from confocal microscopy, thus the simulation results from this study would only represent the peripheral microvasculature or microvasculature grown in window chamber [55, 56], which could be different from the microvasculature in other region of tumor. A second constraint of this investigation was the lack of few key measurements, such as the oxygen consumption rate, thus in simulation they were either derived from appropriated biophysical equation or assigned as constant.

As depicted in Fig. 1, the voxel size is associated with the vascular complexity or average inter vessel spacing and can have a significant impact on the accuracy and precision of the multivariate image-fusion algorithm (MVIF) model. An ideal model would provide a scatter plot with a flat r^2^ and a slope of 1.0 for all voxel sizes. As shown in Fig. 5b, this is clearly not the case, where an optimal voxel size was observed for the tumor and potentially the brain. Visually, this appears to depend on the voxel size relative to the inter vessel spacing or vascular density (see Fig. 3). For voxel sizes ranging from 100 to 200 μm, the coefficient of determination (r^2^), reached a maximum and remained relatively flat. For relatively small voxel sizes (50 μm), the average oxygen diffusion length and inter vessel spacing can exceed the voxel’s dimensions. As a result, two outlier groups were introduced into the scatter plot of MPO2 versus GPO2 for the tumor: an overestimated group, of which MPO2 was exceedingly greater than GPO2, and the underestimated group. Since these outliers were not observed for the 50 µm voxel data in the normal brain tissue, it demonstrates the impact of the abnormal and heterogeneous structure and function of the tumor vasculature on these results. To correct for these outliers, an algorithm was developed to account for oxygen diffusion from neighboring voxels (Fig. 6). For relatively larger voxel sizes, the precision and accuracy of MPO2 became worse and the slope (thus sensitivity) decreased; thus, the single vessel model can no longer adequately represent the complexity or heterogeneity of the vasculature, and as a result, the uncertainty in the average pO_2_ in a voxel becomes unacceptable. Given that the inter vessel spacing ranged from ~12 to 125 μm (Fig. 3), consistent with other tumor models (K, L, M), and was acquired within the periphery or rim of the tumor (Fig. 2), the results from this study show that the proposed modified Krogh image fusion model is accurate and precise for voxel sizes 200 μm and below (r^2^ < 0.75 and slope < 0.98). Therefore, the spatial resolution needed will need to be 200 μm or less. Such resolution can be obtained by many small animal imaging systems (photoacoustic, micro-CT, micro-MRI).

The hypoxic fraction (HF) indicates the hypoxic status of the entire tumor, a parameter used in 2-nitroimidazole based immunohistochemistry. Figure 7 showed the HF correlation of MPO2 and the referenced GPO2 with thresholds at 16, 18, 20, and 22 mmHg, a close regression was observed which shows that MVIF can accurately and precisely predict HF as a result of the normal microvasculature. In Fig. 8, the tumor HF data with threshold from 2.5 to 15.0 mmHg with 2.5 mmHg increment were linearly distributed; the linear regression analysis showed that the r^2^ at all voxel sizes was significant except at 300 µm albeit the HF from MVIF was consistently lower than that from Green’s function, and the regression offset was higher than that of brain microvasculature. Abnormal tumor microvessel structure or hemodynamics or both contributed to the HF offset and accuracy.

The oxygen metabolic rate, the blood flow perfusion, and hemoglobin concentration were three dominant factors in tissue oxygen transportation, as displayed in Fig. 9. Although the direct hemoglobin measurements and oxygen metabolic rates were not available in this simulation investigation, we chose Hill’s equation and Michaelis–Menton equation to estimate the hemoglobin concentration and oxygen metabolic rate in MPO2 calculation. For future in vivo experiments monitoring tumor pO2 fluctuations as function of space and time, all three parameters should be measured to achieve accurate and precise MPO2 estimation using MVIF. PCT-S is capable to derive hemoglobin concentration and oxygen saturation in high spatiotemporal resolution, the oxygen consumption rate can be derived from the fraction of cell volume from DCE-CT or dynamic contrast enhanced photoacoustic tomography. The use of photoacoustic imaging alone can avoid coregistration with DCE-CT, and the concern of radiation superimposing on the tumor progression or compound treatment.

The influence of measurement error of functional imaging to the MPO2 precision and accuracy was simulated and summarized in Fig. 10. Overall, 20 % error (FWHM) in perfusion or fractional vessel volume did not deteriorate accuracy and precision when at least 10 measurements were acquired at each voxel and used to estimate the pO_2_.

There remains limitations of this technique as well as some potential future directions. A key limitation of the proposed technique is for larger voxel sizes. If a model can be devised to rescue the MPO2 versus GPO2 correlation beyond 200 μm, the ability to translate into the clinic is viable.

Multi-vessel models consisting of 2 and 3 parallel cylindrical vessels with identical dimensions and hemodynamics and uniformly spaced within 250 and 300 µm voxels were simulated. However, the improvement was limited. Introducing symmetry into these models and simple bifurcated structures could explain the scatter and nonlinearity in the data; however, identifying or imposing a single concept or methodology would challenging, and requires additional constraints, such as including angiogenic information or inferring a scaling factor based on fractals [57, 58]. Additional pathological features affecting oxygen transport should also be considered, such as oxygen permeability across the vessel wall. Even though tumor vasculature is highly fenestrated and the pressure gradient across the vessel wall reduced due to elevated interstitial fluid pressure, models including oxygen permeability suggest this may have a non-negligible effect [59, 60]. With the ability to measure the permeability-surface area product and interstitial fluid pressure through in vivo imaging, it may be possible to test these models [46, 61]. Green’s function solution of oxygen transport has a number of advantages in the way it handles boundary conditions, the ability to handle a larger set of physiological parameters, efficiency of calculation, and validation to in vivo measurements, it lacks the ability to handle necessary advanced features FDTD and FEM methods support, such as dynamic changes in its parameters and oxygen permeability.

## Conclusion

The results from this simulation study demonstrate that the fusion of in vivo functional imaging based on the MPO2 model to quantify local tumor oxygen concentrations is feasible. A significant correlation was measured between the MPO2 model, a single vessel model, and the Green’s function algorithm (GPO2), a detailed microvascular model, for voxel sizes ranged from 50 to 200 μm, where MPO2 was calculated based on in vivo functional imaging measurements. This upper limit is consistent with the spatial resolution from existing small animal scanners, and provides a new technique to assay in vivo intra-tumor hypoxia. Future work will investigate the potential to monitor the dynamic changes in pO_2_. This additional capability will be able to characterize regions of the tumor undergoing chronic and acute forms of hypoxia and the hemodynamics parameters responsible, thus providing critical information on the role hypoxia and metabolism play in cancer progression and therapy, in particular anti-angiogenic therapies [10, 62].

## Additional file

10.1186/s12938-016-0235-5 Additional data.

## Abbreviations

DCE-CT: dynamic contrast-enhanced computed tomography
PCT-S: photoacoustic computed tomographic spectroscopy
pO_2_: oxygen partial pressure in mmHg
C_tHb_: hemoglobin concentration
SaO_2_: oxygen saturation
GPO2: average pO_2_ derived in a finite volume using Green’s function method
MVIF: multivariate image–fusion algorithm
MPO2: average pO_2_ derived in a finite volume using MVIF model
Rsq or r^2^: R-squared value
