# Supplementary material for: Development of a mathematical model to estimate intra-tumor oxygen concentrations through multi-parametric imaging
Source: Biomed Eng Online. 2016 Oct 12;15:114. doi: 10.1186/s12938-016-0235-5 (PMC5062945; doi:10.1186/s12938-016-0235-5)
Supplement: Supplementary file 1 — 10.1186/s12938-016-0235-5 Additional data. [file 12938_2016_235_MOESM1_ESM.docx]

**Supplemental Data**

**Derivation of multivariate image fusion model for oxygen concentration**

1. Governing principles

The multivariate oxygen transportation model, or the modified cylindrical Krogh oxygenation model, shares many assumptions and governing principles [1], one of which includes that the oxygenation within the tumor is in steady-state. For the purposes of this study, this also requires the image acquisition time needed to obtain functional image data is small compared to temporal changes in the tumor’s hemodynamic properties. Therefore, the oxygen distribution within the tumor is dominated by diffusion, where the oxygen partial pressure denoted by $pO_{2}^{T}(r,z)$ satisfies

$$D\nabla^{2}pO_{2}^{T}=HM\left(pO_{2}^{T}\right)\text{ where }M(\mathrm{y})\text{ = }\frac{M_{0}\text{ y}}{p_{0}+\text{ y}}$$

$D$is oxygen diffusion constant; $H$is the Henry’s constant; $M$ is tissue oxygen consumption rate, which follows Michaelis-Menton kinetics with maximum oxygen consumption constant$(M_{0})$; and $(p_{0})$is the pO_2_ at half-maximal consumption constant. The solution to the above diffusion equation in cylindrical coordinate system is

$D\left[\frac{1}{r}\frac{\partial}{\partial r}\left(r\frac{\partial pO_{2}^{T}}{\partial r}\right)\right]=HM\left(pO_{2}^{T}\right)$ (1)

where r represents the radial distance from the center of the cylinder, z denotes the vessel length along the central axis of cylinder, and terms with a second derivative on z were eliminated. Within the blood vessel, oxygen is either dissolved in plasma or bound to hemoglobin within the red blood cells. In MPO2, the oxygen in the blood vessel is assumed to be carried by hemoglobin (more than 90%), and the regulation of the bound oxygen to the amount of dissolved oxygen within in blood vessel is controlled by the plasma pO_2_ as described by the dissociation curve. Thus the dissolved oxygen concentration in plasma,$C\left(r,z\right)$, is formulated as function of the diffusion rate (D), the blood flow (F), and the oxygen released from hemoglobin,

$$F\frac{\partial C}{\partial z}=D\left[\frac{1}{r}\frac{\partial}{\partial r}\left(r\frac{\partial C}{\partial r}\right)+\frac{\partial^{2}C}{\partial z^{2}}\right]+R$$

In the above equation, R is the deoxygenated hemoglobin production rate, which is proportional to the dissolved oxygen concentration in plasma. The saturated hemoglobin concentration$C'\left(r,z\right)$bound to hemoglobin in the RBCs is represented in following equation:

$$F\frac{\partial C'}{\partial z}=R'$$

where R’ is the oxygenated hemoglobin production rate (negative of R). Since Hill’s equation describes the interaction of the dissolved oxygen to the oxygen bound to hemoglobin, the above two differential equations can be combined into the following equation:

$F(1+m)\frac{\partial pO_{2}^{B}}{\partial z}=D\left[\frac{1}{r}\frac{\partial}{\partial r}\left(r\frac{\partial pO_{2}^{B}}{\partial r}\right)\right]\text{ where }m=nP_{50}^{n}H\lambda_{sat}\frac{(pO_{2}^{B})^{n-1}}{\left(P_{50}^{n}+(pO_{2}^{B})^{n}\right)^{2}}$ (2)

$pO_{2}^{B}$is the oxygen partial pressure in blood which controls the oxygen release from hemoglobin;$\lambda_{sat}$is the saturated hemoglobin concentration;$n$represents the average oxygen bound to hemoglobin; $P_{50}^{n}$is the partial oxygen pressure when oxygen saturation(SaO_2_) is equal to 50%. At the boundary between the vessel wall and tissue, the oxygen partial pressure and its first derivative in equation (1) and (2) is presumed to be continuous:

$$pO_{2}^{B}=pO_{2}^{T}\text{=}pO_{2}\text{ and }\frac{\partial pO_{2}^{B}}{\partial r}=\frac{\partial pO_{2}^{T}}{\partial r}=\frac{\partial pO_{2}}{\partial r}\text{ ; at }r=r_{c}$$

The final expression for pO_2_ within the region of interest is therefore expressed:

$pO_{2}(r,z)=pO_{2}(z=0)-\frac{M\cdot H}{(1+m)F}G_{2}(z;r_{c},r_{T})-\frac{M\cdot H}{2D}G_{1}(r;r_{c},r_{T})$ (3)

where

$$G_{1}(r;r_{c},r_{T})=\frac{r_{c}^{2}}{2}\left(1-\left(\frac{r}{r_{c}}\right)^{2}\right)-r_{T}^{2}\cdot\ln\left(\frac{r}{r_{c}}\right)$$

and

$G_{2}(z;r_{c},r_{T})=\left[\left(\frac{r_{T}}{r_{c}}\right)^{2}-1\right]\cdot z$ (4)

The initial pO_2_ value is function of SaO_2_ which can be measured by PCT-S.

2. Algorithm implementation

All algorithms were programmed in MATLAB (Mathworks, Inc., MA, USA). The size of voxel grid was first determined and randomly superimposed over the vessel network; followed by the effective vascular inputs and the corresponding MPO2 calculation. In this study, three vascular inputs were determined from the available vascular data and described below.

MPO2 vessel radius

A stereological method was used to calculate the total vessel volume within a voxel based on the vessel segments. Briefly, a vessel segment was discretized into a set of points uniformly distributed within the vessel’s cylindrical coordinate system, where the sampling rate along r- and z-axis occur every 1 μm and the angular sampling occurred every 5 degrees. The fraction of the vessel volume within each voxel was determined by the fraction of these points falling within the volume of the voxel. This method is not only very accurate but accounts for the volumetric contribution for those vessel segments that clip the corner or edge of a voxel [2]. To convert the combined vascular volume of the microvasculature to an equivalent radius (r) for a single MPO2 cylindrical vessel segment, the cylindrical volume formula was implemented (vascular volume = πr^2^L), where the length of the vessel (L) is equal to the length of the voxel.

MPO2 blood flow

The micro vascular blood flow in this theoretical investigation is assumed to be uniform in each vessel segment and follows mass conservation at every branch point. Blood flow into or out of a vessel was calculated normal to the cross sectional surface. The magnitude of the blood flow within a voxel was calculated from all the vessel segments crossing within the voxel. Note that blood perfusion (blood flow/volume) is usually the output of dynamic contrast-enhanced imaging modalities, such as DCE-CT and DCE-MRI. The conversion of blood perfusion to blood flow can be achieved by multiplying the voxel volume before inserting in the equation or replacing the blood flow with blood perfusion by dividing the voxel volume in the oxygen transportation equation.

Initial voxel pO_2_ calculation

A Krogh cylindrical model requires the initial or input pO_2_ as a determinant to calculate the pO_2_ distribution within the voxel. Most simulations implementing a Krogh-type of model adopted a physiological pO_2_ as the initial pO_2_ value and used it as a constant in their studies. Often the choice of the initial pO_2_ varied among publications. In this study, the intravessel pO_2_ as determined by Green’s function was used to determine the average SaO_2_ in a voxel as measured from *in vivo* imaging, which in turn, was used to determine the initial pO_2_. The latter was used as an input parameter to the MPO2 model. The oxygen saturation level within a microvessel segment (determined from confocal microscopy) was calculated using the intravessel pO_2_ value and by inverting Hill’s. The volume-weighted average SaO_2_ value within a voxel was calculated with the following formula:

$$SaO_{2}(voxel)=\frac{\sum_{i} S_{i}\bullet V_{i}}{\sum_{i} V_{i}}$$

S_i_ is the SaO_2_ of the i^th^ vessel segment, and $V_{i}$is the volume of the blood vessel inside the voxel from this same vessel segment. The initial pO_2_ at the entrance of the voxel in the MPO2 model was set to be twice the voxel pO_2_. The rationale is as follows.

Given that this equation is linear along the z-axis (length of the blood vessel), the relationship between the average pO_2_ and input pO_2_ is

$$pO_{2}(z)=pO_{2}(z=0)-\frac{MH}{\left(1+m\right)F}\left[\left(\frac{r_{T}}{r_{c}}\right)^{2}-1\right]z$$

which can be rewritten as

$$\left\langle pO_{2}\right\rangle=\frac{pO_{2}(0)+pO_{2}(L)}{2}$$

or

$$\left\langle pO_{2}\right\rangle=pO_{2}(0)+pO_{2}(L)=2\cdot pO_{2}(0)+\frac{M\cdot H}{(1+m)F}G_{2}(r_{c},r_{T})\cdot L$$

The second term on the right is a constant; therefore,

$$pO_{2}(0)=\frac{1}{2}\cdot\left(\left\langle pO_{2}\right\rangle-\frac{M\cdot H}{(1+m)F}G_{2}(r_{c},r_{T})\cdot L\right)$$

However, an exception occurs if pO_2_ is less than zero along the length of the blood vessel. In this situation, the resulting negative values are set to zero and a recalculation of pO_2_(0) is determined.

**References**

1. Fournier, R.L., *Basic Transport Phenomena in Biomedical Engineering*. 3rd ed. 2011: CRC Press.

2. Mouton, P.R., *Unbiased Stereology*. 2011: The Johns Hopkins University Press.
